# Effect of liquefaction temperature and enzymatic treatment on bioethanol production from mixed waste baked products

**DOI:** 10.1186/s12896-025-01037-6

**Published:** 2025-09-08

**Authors:** Mervat Almuhammad, Ralf Kölling, Daniel Einfalt

**Affiliations:** 1 Yeast Genetics and Fermentation Technology, Institute of Food Science and Biotechnology, University of Hohenheim, Garbenstraße 23, 70599 Stuttgart, Germany; 2 Botanical Garden, Ulm University, Hans-Krebs-Weg, 89081 Ulm, Germany

**Keywords:** Food waste, Fermentation, Bioethanol, Liquification enyzme, Saccharification enyzmes

## Abstract

This study investigates the effect of different liquefaction temperatures (50–70 °C) and four commercial enzyme formulations on glucose release and subsequent ethanol yield, using mixed waste baked products as a substrate. Among the enzymes tested, Amylase GA 500 proved to be superior in the hydrolysis of starch at lower temperatures (50 °C and 55°C). At higher liquefaction temperatures (65 °C and 70°C) all four enzyme preparations showed comparable activity. The highest glucose concentration (205.7 g/L) and the highest ethanol yield (92 g/L) were achieved with Amylase GA 500 at 65 °C. Its superior performance is attributed to the synergistic activity of α-amylase and glucoamylase, which facilitates efficient starch hydrolysis. Crucially, we discovered that the liquefaction temperature profoundly affects fermentation speed independently of the initial glucose concentration or the enzyme preparation used for starch hydrolysis. This novel mechanistic insight suggests that higher temperature treatment either makes an additional factor crucial for yeast fermentation available or depletes/destroys an inhibitor present in the complex waste bakery product matrix. These findings highlight the critical role of temperature and enzyme formulation in optimizing bioethanol production from bakery waste, supporting the development of more sustainable and efficient waste-to-biofuel processes.

**Graphical Abstract:**

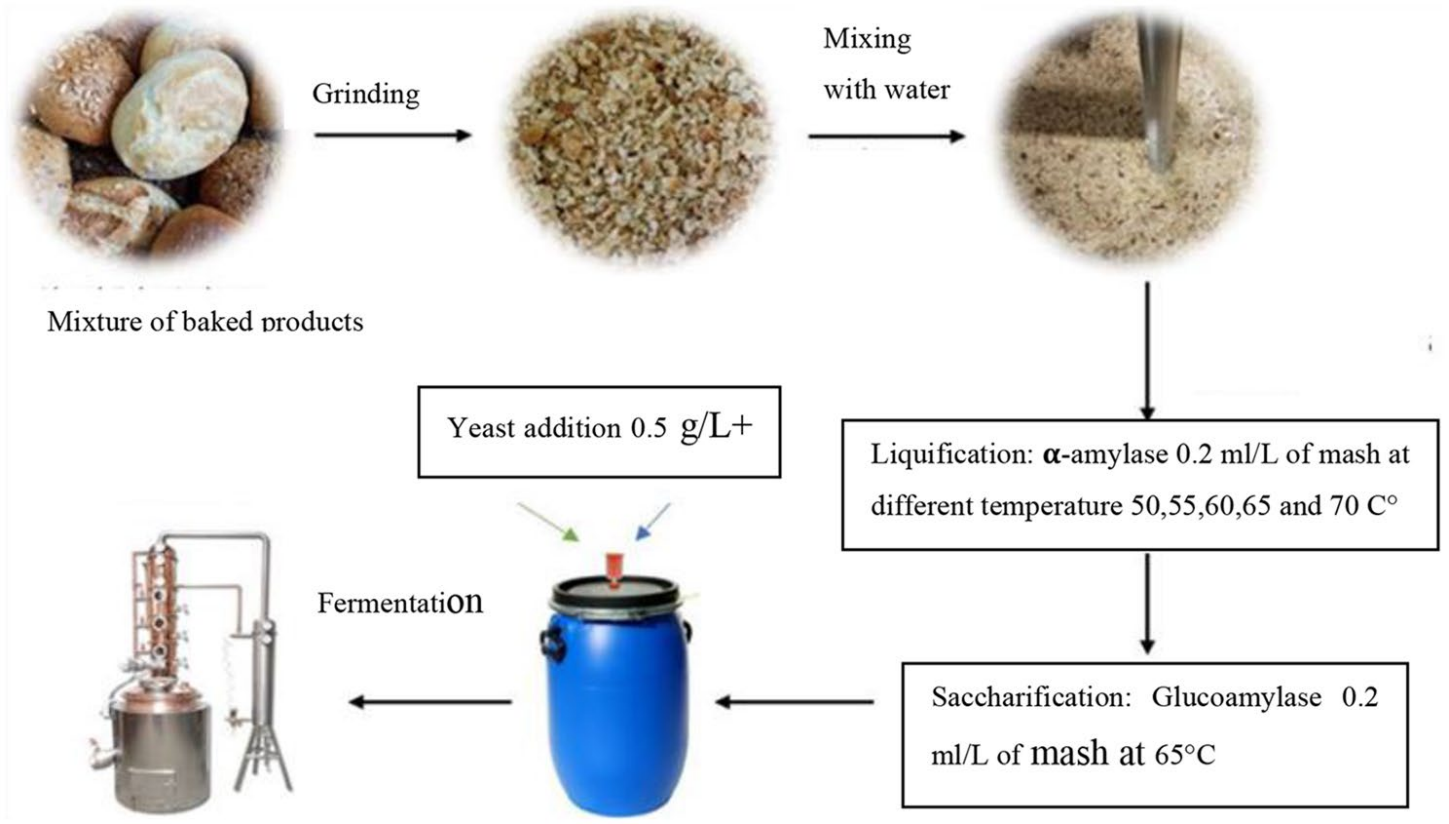

## Introduction

Reducing food waste and establishing efficient waste management systems are among the most pressing challenges facing the modern world. Currently, around one-third of all food produced globally is wasted, resulting in the loss of critical resources such as energy, freshwater, and arable land. Inadequate waste handling further contributes to greenhouse gas emissions, including carbon dioxide, which accelerates global warming [1].

The baking industry is particularly affected by this issue. Due to the widespread consumption of bread, global production has reached nearly 100 million tons per year, generating substantial amounts of waste [2]. It is estimated that 7–10% of bakery products are lost annually throughout the supply chain. This represents a significant problem, especially in Europe, which accounts for over 53.6% of global bread production [3]. In the UK alone, approximately 290,000 tons of bakery waste are generated annually, contributing to both environmental degradation and economic loss. Sweden also faces a major challenge, with households producing around 29,870 tons of bakery waste annually, and the entire supply chain generating nearly 80,410 tons [4]. Similar trends are observed elsewhere: in the United States, around 20% of bread is wasted in households and 12% by retailers, while in Germany, approximately 14% of bakery waste occurs during the production process. In Poland, an estimated 170,000 tons of bakery waste are produced each year [5].

Bakery waste contains numerous valuable components, including carbohydrates (mainly starch), proteins, and fats, which offer considerable potential for use in biotechnological processes [6]. However, the growing volume of this waste necessitates the adoption of efficient management strategies, guided by regulatory policies and circular economic principles. Major sources of bakery waste include overproduction and the inability to sell products past their expiration date. Additionally, the use of contaminated or defective materials during production contributes to further waste. The issue is compounded by the rapid staling and spoilage of bakery goods, which significantly increases the overall waste generated [5].

Bread waste (BW), characterized by its high content of over 70% starch, along with 8–10% protein and 1–5% fat [7], serves as an excellent substrate for bioethanol generation through anaerobic fermentation by *Saccharomyces cerevisiae*. This conversion process initiates with the hydrolysis of starch to liberate fermentable sugars. Although dilute acid hydrolysis effectively cleaves glycosidic bonds, enzymatic hydrolysis is generally favored due to its cleaner product profile and the absence of inhibitory by-products [8]. A typical enzymatic approach for starch conversion often encompasses three primary stages: an initial gelatinization phase, commonly around 105 °C for 5 minutes; a subsequent liquefaction step, typically at 95 °C for 2 hours with α-amylase; and finally, saccharification, usually performed at 50–60 °C over 48–72 hours using glucoamylases [9].

In our previous study on six typical German waste baked products, we demonstrated their substantial potential for bioethanol production, using a liquefaction temperature of 90 °C and a saccharification temperature of 65 °C to maximize glucose release [10]. While our previous study [10] explored higher liquefaction temperatures, including 90∘C, the current study specifically aimed to investigate the potential for energy savings by focusing on a lower temperature range (50 − 70∘C). So far, most studies have applied high-temperature pretreatments for starch hydrolysis. However, as baked products are already subjected to high heat and processing during production, the starch they contain may be more readily hydrolyzed, potentially enabling effective saccharification at lower temperatures. In this study, we systematically investigated the effect of different liquefaction temperatures and enzymatic treatments on glucose release and subsequent ethanol production from a heterogeneous mixed sample of waste baked products. Our aim was not only to determine if comparable or improved results could be achieved under lower, energy-saving temperature conditions but also to uncover novel insights into the complex interplay between pretreatment, enzyme performance, and fermentation kinetics for this challenging, real-world substrate.

## Materials and methods

### Leftover baked products

The substrate for this investigation comprised a blend of six distinct types of one-day-old leftover baked products obtained from Webers Backstube bakery (Friedrichshafen, Germany). This mixture, consisting of bread rolls (Brötchen), pretzel rolls (Laugengebäck), fine rye bread (Mischbrot), white bread (Weißbrot), pastry (Plunder), and cream cakes (Sahne-Cremetorten), was prepared to represent typical bakery waste. Further details on the specific products and their ingredients are provided in Tables 1 and 2. Proximate analysis for crude fat and protein content was conducted by the Core Facility Hohenheim (CFH), as described in our prior work [10].

**Table 1:** Overview of the different leftover baked product groups with their baking ingredients

Sample groups	Overview of ingredients
Bread rolls	Wheat flour 550, rye flour 1150, poppy seeds, grains, salt, caraway seeds
Pretzel rolls	Wheat flour 550, milk powder, butter, pretzel lye
Fine Rye Bread	Wheat flour 550/1050, rye flour 1150
White Bread	Wheat flour 550/1050.
Pastry	Wheat flour 550, butter, milk powder, honey, egg, cherries.
Cream Cakes	Wheat flour 550, egg, sugar, butter, quark, whipped cream, corn starch, fruits.

**Table 2:** Sample compositions of waste baked products used in this study

Sample groups	Dry matter (DM) [%FM (fresh matter)]	Organic dry matter (oDM) [%DM]	Crude Fat [%DM]	Protein [%DM]
Bread rolls	75.94	97.98	2.62	11.38
Pretzel rolls	82.19	97.66	4.32	12.15
Fine Rye Bread	60.17	97.84	2.20	8.60
White Bread	74.57	98.43	2.29	9.94
Pastry	66.05	99.09	17.75	6.94
Cream Cakes	58.84	99.00	20.72	6.50
Mixed baked	64.23	98.02	-	-

### Mash preparation

Mash preparation followed a protocol similar to our previous study [10], with specific adaptations for the current investigation. A representative substrate was formed by combining equal weights of the six aforementioned baked products. This blend was then comminuted to particles smaller than 7 mm using a Vorwerk Deutschland Stiftung & Co. KG thermal mixer (Wuppertal, Germany). Prior to each experiment, the ground mixture, stored at −18 °C, was brought to 20 °C. For each trial, 100 ± 2 g of the mixed product (64.23% dry matter, as detailed in Table 2) was combined with 114.1 g of water to achieve a final mash with 30% dry matter. The resulting mixture was gently agitated until a uniform consistency was achieved.

### Enzymatic treatment

This study specifically focused on developing a simple and fast practical method for the conversion of starch from raw, heterogeneous mixed bakery waste, which represents a methodological contribution for industrial waste valorization. For the enzymatic hydrolysis, four distinct α-amylase preparations were chosen for the liquefaction phase, selected based on their origin, enzymatic activity, and relevance in industrial applications (Table 3). These included EnerZyme® AMYL, Still Spirits Alpha-Amylase, Amylase GA 500 (all from ERBSLÖH Geisenheim GmbH, Germany), and Schliessmann-VF Enzym (C. Schliessmann Kellerei-Chemie GmbH & Co. KG, Germany), all commercially available and utilized in brewing, bioethanol, and baking industries. The enzymatic process involved two sequential stages: liquefaction and saccharification. This sequential approach was chosen because preliminary experiments exploring simultaneous addition of both α-amylase and glucoamylase did not yield superior results and, in some cases, led to reduced efficiency, likely due to the differing optimal pH and temperature conditions for the individual enzymes (e.g., α-amylase optimal pH 6.5, glucoamylase optimal pH 4). Liquefaction was conducted by adding 0.2 mL/L of one of the selected α-amylase preparations to the mash, with the process carried out at five different temperatures (50 °C, 55 °C, 60 °C, 65 °C, and 70°C°C) for 30 minutes under continuous agitation in a thermal mixer. Following liquefaction, saccharification was performed using 0.2 mL/L of glucoamylase EnerZyme® HT (ERBSLÖH Geisenheim GmbH, Germany) at 65 °C for 30 minutes with continuous stirring, converting the liquefied starch into fermentable sugars. The 30-minute liquefaction time was deemed sufficient due to the pre-gelatinized nature of starch in baked products, which makes it more readily accessible to enzymatic hydrolysis compared to raw starch, as evidenced by the high glucose concentrations achieved (up to 205.7 g/L). Mash pH was maintained at optimal levels for each enzyme: pH 6.5 for α-amylase and pH 4 for glucoamylase. Enzyme dosages were determined according to manufacturer guidelines. The consistent dosage of 0.2 mL/L for all α-amylase preparations was based on manufacturers’ recommended guidelines for their respective industrial applications, allowing for a direct comparison of their practical efficacy under industrially relevant conditions rather than normalizing to specific activity units.

**Table 3:** Comparison of enzyme source, activity, and relevance for starch hydrolysis and ethanol production

Enzyme	Source	Activity	Relevance
G	Blend of α-amylase and glucoamylase from microbes	High starch hydrolysis efficiency; glucose production (500 AGU/g (approx. 500 AGU/mL))	Industrial bioethanol production and bread waste valorization
E	Microbial source optimized for rapid starch breakdown	Breaks down starch into oligosaccharides; moderate glucose conversion (Not explicitly stated in standard units)	Used in bioethanol and fermentation processes
P	Microbial, optimized for brewing and distillation	Efficient breakdown of starch into oligosaccharides (Not explicitly stated in standard units)	Widely used in brewing and alcohol production
S	Microbial α-amylase, optimized for starch hydrolysis	Converts starch into oligosaccharides; moderate glucose yield ( > 40.000 RAU/ml)	Common in large-scale starch hydrolysis, especially in baking

Enzymes are represented by the following symbols: G = Amylase GA 500; E = EnerZyme® AMYL; p = Still Spirits Alpha-Amylase; S = Schliessmann-VF Enzyme

The listed activities and applications are based on product information from manufacturers and general knowledge of microbial α-amylases

For EnerZyme® AMYL and Still Spirits Alpha-Amylase, specific activity in standard units was not explicitly provided by the manufacturers; therefore, their practical efficacy was compared based on the recommended industrial dosage.

### Fermentation

Fermentation was conducted using the industrial yeast strain *S. cerevisiae* Ethanol Red® (Lesaffre, France), selected for its established efficacy in ethanol production, high ethanol tolerance, and robust performance under various conditions, ensuring optimal alcohol yields and cell viability. Each fermentation was carried out in 100 mL Erlenmeyer flasks containing 100 mL of the saccharified mash. Yeast was inoculated at a concentration of 0.5 g/L, aligning with the supplier’s recommended range of 250–500 g/m^3^, which translates to an initial cell density of approximately 5–10 million viable cells per milliliter. To enhance yeast metabolic activity and overall fermentation efficiency, diammonium phosphate (DAP) was added to all flasks at a concentration of 300 mg/L. The fermentation process proceeded for 96 hours at 30 °C on a rotary shaker operating at 100 rpm. The pH in the fermentation was 4, a condition resulting from the saccharification step, which is within the optimal range for glucoamylase and suitable for *S. cerevisiae*. It is important to note that the fermentation pH was not continuously monitored throughout the 96-hour period, which is an area for future optimization, especially given potential shifts from the initial acidic pH resulting from saccharification. All experimental runs were performed in triplicate to ensure data reproducibility.

### Analytical methods

To monitor the fermentation process and evaluate sugar-to-ethanol conversion efficiency, samples were collected at 0, 24, 48, 72, and 96 hours. Key metabolites, including glucose, ethanol, glycerol, acetic acid, and lactic acid, were quantified. Sample preparation involved centrifuging each mash sample at 10,000 rpm for 10 minutes to remove solids, followed by filtration of the supernatant through 30 mm qpore membrane filters (0.45 µm pore size). The resulting filtrate was then transferred to 2 mL neochrom® vials and diluted 1:5 with bi-distilled water. Analysis was performed using a High-Performance Liquid Chromatography (HPLC) system (1260 Infinity II, Agilent, Santa Clara, USA), equipped with a 1260 Infinity II G7129A autosampler. Chromatographic separation utilized a Rezex ROA-Organic Acid H^+^ (8%) polar column (Phenomenex Inc., Torrance, CA, USA). A 20 µL aliquot of each sample was injected. The mobile phase consisted of 0.5 M sulfuric acid, delivered at a flow rate of 0.6 mL/min, with a maximum operating pressure of 100 bar. The column temperature was maintained at 40 °C. Comprehensive instrument settings are provided in Table 4. Chromatographic data were acquired using OpenLab software and subsequently processed in Microsoft Excel for evaluation, consistent with our prior analytical approaches [10].

**Table 4:** HPLC analysis operating conditions and parameters

	Value	Unit
RID		
Column temperature	40	°C
Column dimensions	50 ×7.8	mm
Polarity	positiv	
Column oven temperature	80	°C
Quaternary pump (G7111A)		
Eluent flow rate	0.6	mL/min
Pressure	71.74	bar
Pressure Limit	100	bar

### Statistical analysis

To evaluate the effects of enzyme type and liquefaction temperature on ethanol production, a two-way analysis of variance (ANOVA) was performed using the PROC GLM procedure in SAS (version 9.4, SAS Institute, Cary, NC, USA). The analysis examined the main effects of the liquefaction enzyme (EnerZyme® AMYL, Still Spirits Alpha-Amylase, Amylase GA 500, Schliessmann-VF Enzym), the temperature (50 °C, 55 °C, 60 °C, 65 °C, 70 °C), and the interaction between enzyme and temperature on ethanol concentration. Statistical significance was assessed at a 95% confidence level (*p* < 0.05).

## Results and discussion

### Glucose concentration after enzymatic treatment

**Fig. 1:**
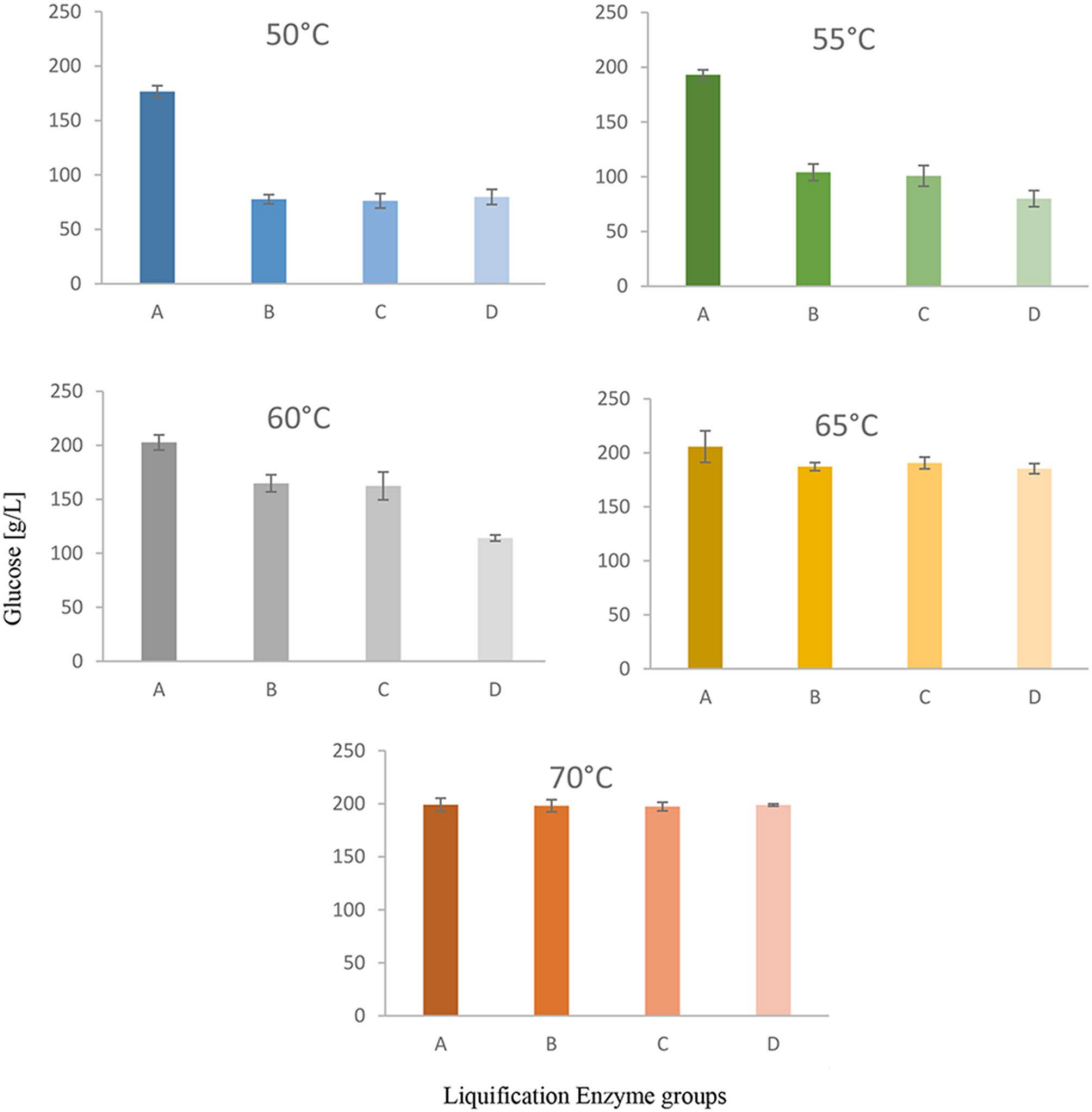
Mean glucose concentrations in mashed baked product mixtures after liquefaction using four different enzymes: a – amylase GA 500, B – EnerZyme® AMYL, C – Schliessmann-VF enzym, and D – Still spirits alpha-amylase, at varying temperatures, followed by saccharification with glucoamylase (EnerZyme® HT). Values are means ± standard deviation (SD), *n* = 3. Temperatures as indicated

The four enzyme preparations differed markedly with respect to their temperature dependence (Fig. 1). At high temperatures (65 °C and 70°C°C) all four preparations were similarly active giving rise to glucose concentrations between 185.3 and 205.7 g/L. However, at temperatures below 65 °C, there was a clear difference between Amylase GA 500 and the other three preparations. While the Amylase GA 500 activity was only slightly lower (producing between 176.6–202.7 g/L glucose) the activity of the other three preparations was much less. At 50 °C and 55°C°C it was only about half as high as the Amylase GA 500 activity (for exact values see Table 5).

**Table 5:** Ethanol production and conversion efficiency in baked products, obtained using different enzymes at various temperatures. Enzymes are represented by the following symbols: G = amylase GA 500; E = EnerZyme® AMYL; *p* = still spirits alpha-Amylase; S = schliessmann-VF enzyme

Temperature (°C)	Enzyme	Glucose (g/L) ± SD	Ethanol (g/L) ± SD	Theoretical Ethanol (g/L)	Conversion Efficiency (%)
50	G	176.6 ± 5.3	57.09 ± 7.6	90.3	63.3
	E	77.6 ± 4.2	23.18 ± 1.6	39.7	58.9
	P	79.7 ± 6.9	25.9 ± 2.5	40.7	63.9
	S	76.2 ± 6.6	26.0 ± 5.9	38.9	66.8
55	G	193.1 ± 4.5	76.13 ± 2.6	98.7	77.1
	E	104.1 ± 7.5	38.0 ± 6.4	53.2	71.4
	P	80.1 ± 7.4	31.0 ± 2.1	40.9	75.8
	S	100.9 ± 9.4	38.0 ± 4.6	51.6	73.6
60	G	202.7 ± 7.2	83.0 ± 9.4	103.6	80.1
	E	164.8 ± 7.8	68.0 ± 3.7	84.2	80.7
	P	114.1 ± 2.8	48.0 ± 2.9	58.4	82.2
	S	162.4 ± 12.9	67.0 ± 9.3	83.0	80.7
65	G	205.7 ± 14.1	92.0 ± 3.7	105.2	87.5
	E	187.2 ± 3.7	87.0 ± 21.9	95.7	90.9
	P	185.3 ± 4.6	83.0 ± 4.3	94.7	87.6
	S	190.6 ± 5.4	80.0 ± 5.7	97.4	82.2
70	G	199.1 ± 6.1	86.5 ± 8.3	101.7	85.5
	E	198.1 ± 5.7	81.75 ± 6.4	101.2	80.8
	P	198.8 ± 1.1	89.0 ± 0.4	101.6	78.6
	S	197.3 ± 4.1	81.0 ± 0.9	100.8	80.4

This aligns with earlier findings that enzyme blends combining α-amylase and glucoamylase yield superior hydrolytic performance [11]. Amylase GA 500 is a combination of α-amylase and glucoamylase, enabling synergistic action: α-amylase rapidly depolymerizes starch into oligosaccharides, which glucoamylase then converts into glucose. This prevents accumulation of intermediates and avoids product inhibition. In contrast, the α-amylase-only formulations lack this synergy and thus require more extensive gelatinization or higher temperatures to achieve comparable glucose yields.

Additionally, the strong performance of Amylase GA 500 is supported by Liakopoulou-Kyriakides et al. [12], who found that microbial enzyme blends of α-amylase and glucoamylase significantly improve starch hydrolysis, even without prior gelatinization. The thermostability and synergistic efficiency of such blends likely translates into the robust results observed here.

Thus, with Amylase GA500, starch liquification can be performed efficiently at temperatures as low as 50 °C, which would lead to significant energy savings compared to the other three preparations that require higher temperatures for optimal activity. This specific empirical demonstration of a commercial blend’s superior performance under conditions conducive to energy savings for a complex, mixed waste substrate represents a novel and industrially significant finding, providing concrete, actionable data for process optimization.

The glucose concentration obtained under the optimal enzymatic treatment and temperature profile in the current study aligns closely with theoretical estimates. For instance, Zhang and Lynd [13] modelled the theoretical upper limit of enzymatic glucose release from starchy substrates and reported conversion efficiencies in the range of 90–95%, which aligns with the 205.7 g/L achieved using Amylase GA 500 at 65 °C in the present study. In addition, Almuhammad et al. [10] reported a weighted average glucose concentration of 206.3 g/L for an optimally mixed bakery substrate. The present study achieved 205.7 g/L using Amylase GA 500 at 65 °C, confirming the effectiveness of this enzymatic strategy for valorising mixed bread waste. The glucose yields achieved here also demonstrate strong reproducibility and industrial relevance.

The observed temperature-dependent increase in glucose yields is attributed to enhanced enzymatic kinetics and progressive starch gelatinization, which improves substrate accessibility—a trend supported by previous studies on starch structure and enzyme interaction [14]. This trend was consistent across all enzyme systems up to 65 °C.

These findings are consistent with earlier reports. Lineback and Wongsrikasem [15] demonstrated that starch gelatinization levels in baked goods vary widely, influencing enzymatic digestibility. In the present study, increasing glucose yields at higher temperatures likely stem from progressive gelatinization, enhancing susceptibility to enzymatic breakdown.

### Ethanol production during fermentation

**Fig. 2:**
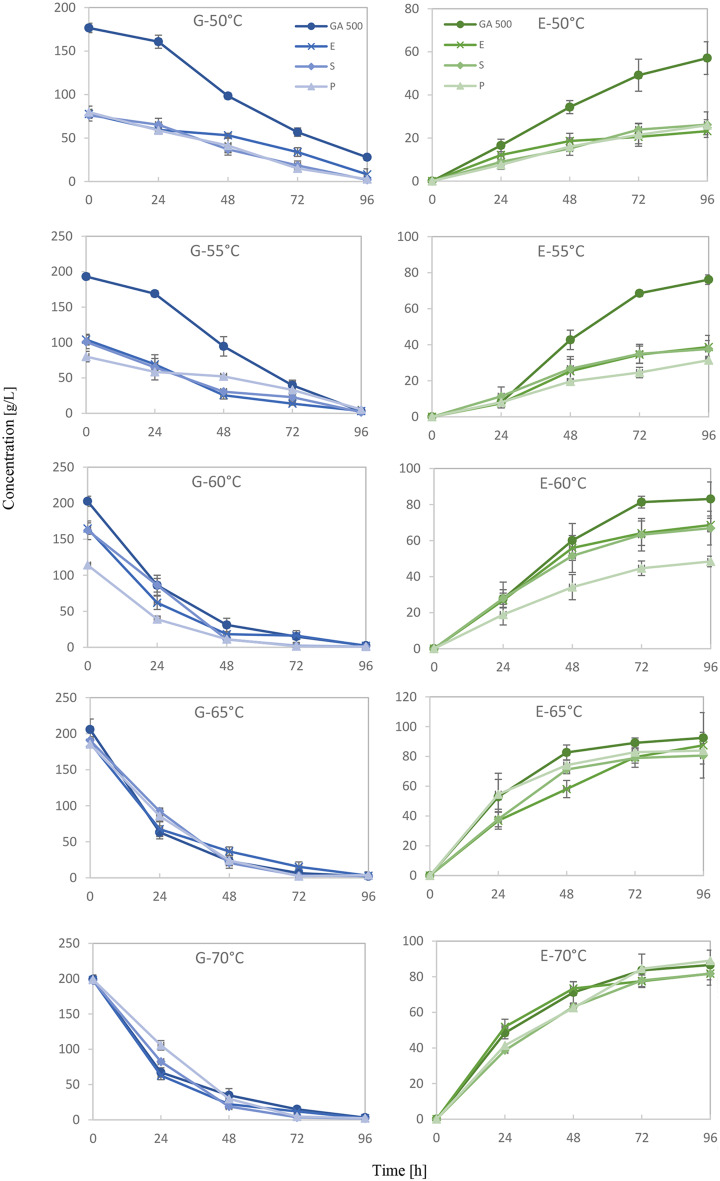
Glucose (G) and ethanol (E) concentrations during fermentation of waste baked products pretreated with four enzymes: GA 500, EnerZyme® AMYL (E), schließmann-VF (S), and still spirits alpha-amylase (P). Liquefaction temperatures used prior to fermentation are indicated. All values represent mean concentrations (*n* = 3) ± standard deviation

In principle, the ethanol concentration obtained after fermentation should be proportional to the glucose concentration present at the beginning of fermentation. If glucose were completely converted to ethanol, then 51% of the mass of glucose should be recovered in form of ethanol. Such high conversion rates are usually not observed, since part of the glucose is also invested into the production of biomass. Basically, this correspondence between initial glucose concentration and ethanol yield was observed in the fermentation experiments (Fig. 2). Conversion rates between 58.9% and 90.9% could be achieved (Table 5). But curiously, it turned out that the conversion rate was dependent on the temperature under which the starch hydrolysis had been performed. The lowest conversion rates of about 60% were obtained with starch hydrolysis at 50 °C and the highest rates close to 90% with hydrolysis at 65 °C, irrespective of the initial glucose concentration in the fermentation and the enzyme preparation used for hydrolysis. From the glucose consumption curves in Fig. 2, it is evident that fermentation proceeded significantly slower with the low-temperature hydrolysates. For instance, at 50 °C, a substantial amount of residual glucose remained at the end of the 96-h fermentation period, whereas glucose was completely consumed after 72 h with the high-temperature hydrolysates. This novel observation indicates that, beyond glucose concentration, another factor crucial for yeast fermentation is either made available by high-temperature treatment of the samples or an inhibitor of yeast fermentation is depleted or destroyed by it. This mechanistic insight warrants further investigation. While higher viscosity and potential fermentation inhibitors from the mixed baked product matrix may have stressed the yeast and reduced ethanol yield [16, 17], the temperature-dependent effect on fermentation kinetics, irrespective of initial glucose levels, points to a more fundamental interaction.

A potential factor in this context could also be the supply of yeast assimilable nitrogen (YAN) in the form of amino acids from the proteolysis of proteins in the sample. Although YAN in form of DAP was added to the mashes, a higher supply of amino acids could further enhance fermentation speed [18]. This needs to be explored in further studies.

Overall, these findings demonstrate that liquefaction temperature strongly influences the fermentation rate and thus ethanol yield [19]. Lower temperatures (50 °C and 55 °C°C) resulted in reduced substrate breakdown, slower fermentation, and lower ethanol output. In contrast, higher temperatures (60 °C and above) improved substrate accessibility and fermentation kinetics, leading to higher ethanol concentrations and improved conversion efficiencies [18].

Although Amylase GA 500 proved to be superior to the other enzyme preparations in starch hydrolysis at lower temperatures, this effect is refuted by the low fermentation efficiencies with low temperature hydrolysates. The highest ethanol yields were obtained with hydrolysates at 65 °C. Under these conditions all four tested enzyme preparations performed equally well. It is important to note that while high glucose concentrations were achieved, there remains a challenge with complete fermentation, potentially due to the viscosity of the mash or other unidentified inhibitors. Further research is warranted to optimize the fermentation process and ensure the complete conversion of all glucose to ethanol, possibly by exploring rheological properties or alternative fermentation strategies.

Statistical analysis confirmed that both temperature and enzyme type had a significant effect on ethanol production (*p* < 0.0001). For each enzyme, ethanol yield increased significantly with rising temperature from 50 °C to 65 °C, indicating strong temperature dependence. Moreover, there were significant differences in ethanol production between enzymes at 50 °C, 55 °C, and 60 °C, confirming that enzyme selection plays a critical role at lower temperatures. However, at 65 °C and 70 °C, the interaction between enzyme type and time was not statistically significant, suggesting that ethanol yields among all enzymes converged—likely due to enzyme saturation or thermal inactivation (see Tables 6 and 7).

**Table 6:** Two-way ANOVA for bioethanol production at different temperatures, showing the interaction effect between enzyme type: G = amylase GA 500; E = EnerZyme® AMYL; *p* = still spirits alpha-amylase; S = schliessmann-VF enzyme

Temperature (°C)	DF	F-Value	p-Value*
50	12	6.83	< 0.0001
55	12	13.65	< 0.0001
60	12	2.40	0.0189
65	12	1.00	0.4658
70	12	1.57	0.1411

< 0.05 significant effect

**Table 7:** Two-way ANOVA on bioethanol production for each enzyme across all temperatures (50 °C to 70 °C), showing the interaction effect between temperature and time. G = amylase GA 500; E = EnerZyme® AMYL; *p* = still spirits alpha-amylase; S = schliessmann-VF enzyme

Enzyme	DF	F-Value	p-Value*
E	16	7.95	< 0.0001
G	16	4.89	< 0.0001
S	16	9.63	< 0.0001
P	16	19.20	< 0.0001

< 0.05 significant effect

Similar to our findings, previous studies have also demonstrated that combining food-grade enzymes can significantly enhance the hydrolysis of bread waste. Rosa-Sibakov et al. [20] reported that the combination of α-amylase and amyloglucosidase resulted in a high glucose yield of up to 93% when recycling waste bread for use in wheat bread-making processes. Compared to previous work focusing on enzymatic hydrolysis of waste bread for sugar recovery and food applications [20], the present study explored the full bioconversion of hydrolyzed bread waste into bioethanol through fermentation. In addition to testing different commercial enzymes for hydrolysis efficiency, the impact of liquefaction temperature on subsequent fermentation performance was systematically evaluated. This approach highlights the potential of waste bread not only for food recycling but also for sustainable biofuel production.

While combining hydrolysis and fermentation into a one-step process (Simultaneous Saccharification and Fermentation, SSF) can offer advantages such as reduced product inhibition and potentially lower costs, it also presents significant challenges. The primary difficulty lies in the differing optimal temperature and pH conditions for α-amylase (liquefaction), glucoamylase (saccharification), and yeast fermentation. For instance, α-amylase was used at pH 6.5 and varying temperatures, while glucoamylase was used at 65 °C and pH 4. Attempting a single-step hydrolysis would necessitate a compromise on these optimal conditions, potentially leading to suboptimal performance for one or more components. Preliminary experiments indeed showed that combining both enzymes simultaneously did not yield good results, thus justifying the chosen sequential approach (Separate Hydrolysis and Fermentation, SHF) to maximize the efficiency of each enzymatic step independently.

**Fig. 3:**
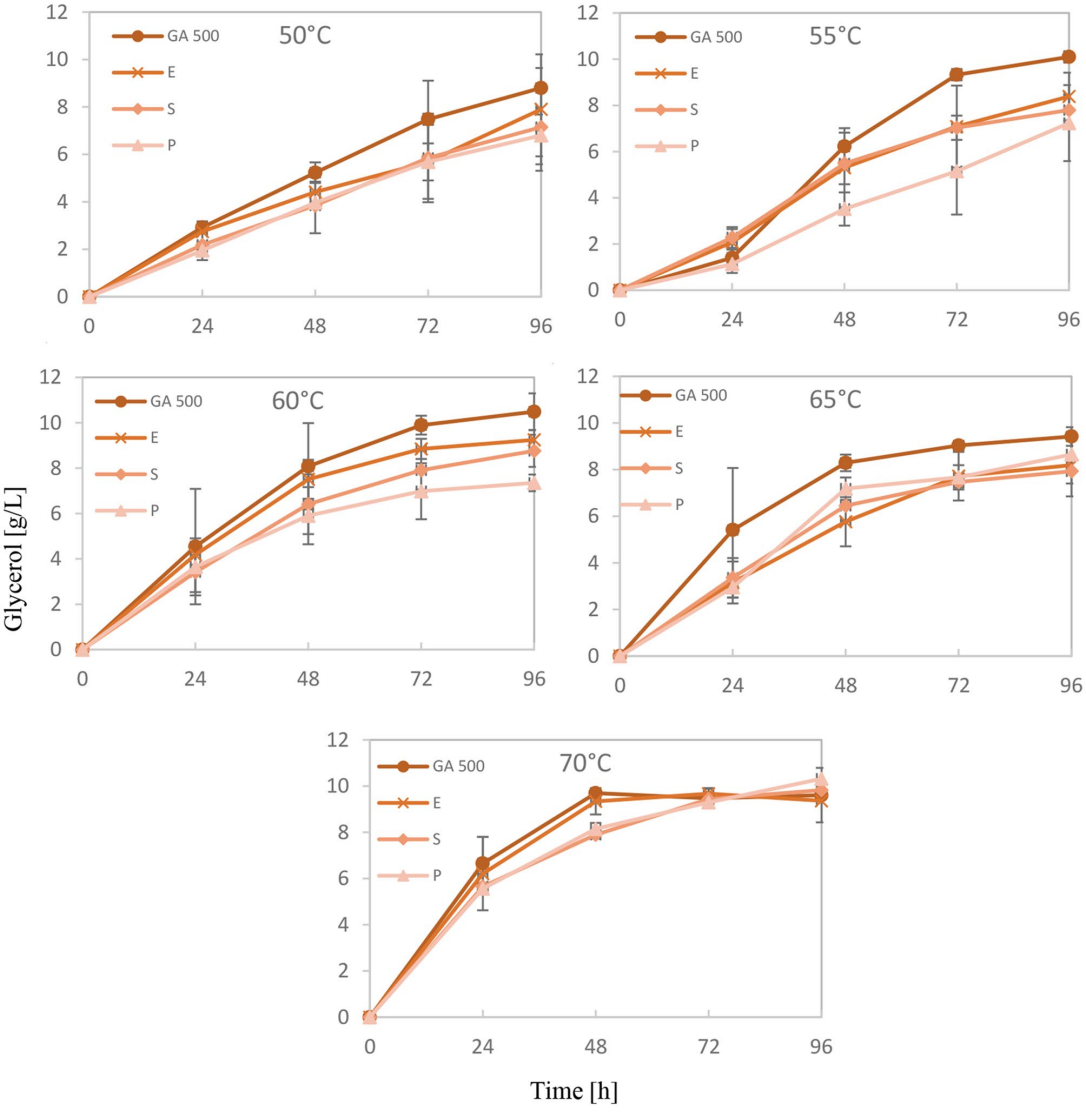
Glycerol concentrations during fermentation of waste baked products pretreated with four enzymes: GA 500, Enzyme® AMYL (E), schliessmann-VF (S), and still spirits alpha-amylase (P). The liquefaction temperatures used prior to fermentation are indicated. All values represent mean concentrations (*n* = 3) ± standard deviation

In addition to ethanol production, glycerol formation was also analyzed as a key fermentation by-product to further understand fermentation dynamics. The low glucose-to-ethanol conversion rate under some conditions could also be due to the production of fermentation by-products instead of ethanol. One such by-product, produced in sizable amounts during fermentation, is glycerol [21]. Therefore, glycerol production was examined in our fermentation experiments (Fig. 3). Overall, glycerol production was largely proportional to the fermentation rate. The hydrolysates treated with Amylase GA 500, which had the highest glucose concentrations—especially at lower liquefaction temperatures—also showed the highest glycerol levels during fermentation. Furthermore, glycerol accumulated more quickly during fermentation of the high-temperature hydrolysates compared to the low-temperature ones; however, the final concentrations ultimately reached were about the same (6–10 g/L).

There was no indication that yeast cells in fermentations with low-temperature hydrolysates suffered from stress conditions such as osmotic pressure or redox imbalance, which would typically lead to elevated glycerol levels [22]. This was not observed. Instead, the reduced glycerol levels at 50 °C likely resulted from slower fermentation rates.

Thus, glycerol production does not appear to be responsible for the differences in glucose-to-ethanol conversion efficiency observed among the various hydrolysates.

## Conclusion

This study evaluated the effect of different liquefaction temperatures on the performance of four commercial enzymes during the hydrolysis and fermentation of waste baked products for bioethanol production. The results demonstrated that liquefaction temperature significantly influenced both glucose release and ethanol yield, with optimal performance observed at 65 °C. Among the enzymes tested, Amylase GA 500 achieved the highest glucose concentration (205.7 g/L) and ethanol yield (92 g/L).

At suboptimal temperatures (50–55 °C), reduced enzymatic activity led to incomplete starch conversion and lower ethanol yields. In contrast, higher temperatures (60–65 °C) enhanced enzymatic kinetics and substrate accessibility, improving overall fermentation efficiency. While final ethanol yields did not significantly increase beyond 65 °C, fermentation kinetics, including the speed of glucose consumption and ethanol production, continued to improve up to 70 °C, as shown in Fig. 2. This indicates that higher liquefaction temperatures can still offer benefits in terms of process efficiency, reinforcing the idea of a beneficial effect of higher temperature pretreatment on yeast performance, even if the final titer plateaus.

These findings highlight the importance of enzyme selection and thermal optimization in improving the efficiency and yield of bioethanol production from starchy food waste. Amylase GA 500 at 65 °C is recommended as the most effective condition for maximizing both saccharification and fermentation outcomes in this process.

## Data Availability

The authors declare that the data supporting the findings of this study are available within the article and from the corresponding author, Mervat Almuhammad, upon reasonable request.

## Abbreviations

ANOVA: Analysis of variance
DAP: Diammonium phosphate
DM: Dry matter
E: EnerZyme® AMYL
G: Amylase GA 500
HPLC: High-Performance Liquid Chromatography
ODM: Organic dry matter
P: Still Spirits Alpha-Amylase
S: Schliessmann-VF Enzyme
SD: Standard deviation
YAN: Yeast assimilable nitrogen
ZIM: Zentrales Innovationsprogramm Mittelstand
